# Editorial: Molecular and genetic mechanisms of chilling tolerance in plants

**DOI:** 10.3389/fpls.2023.1281889

**Published:** 2023-09-07

**Authors:** Baohong Zou, LeiYun Yang, Wenyi Wang, Zemin Zhang

**Affiliations:** ^1^ State Key Laboratory of Crop Genetics & Germplasm Enhancement and Utilization, Nanjing Agricultural University, Nanjing, China; ^2^ Department of Plant Pathology, College of Plant Protection, Nanjing Agricultural University, Key Laboratory of Integrated Management of Crop Diseases and Pests, Ministry of Education, Nanjing, China; ^3^ College of Agriculture, South China Agricultural University, Guangzhou, China

**Keywords:** low temperature, gene expression, GWAS, transcriptome, metabolome

Low-temperature stress poses a great challenge for crop yield and quality (Luo, 2011), which is exacerbated by climate change and global population increase (Fuglie, 2021). Low temperature induced stresses can be classified either as chilling stress or freezing stress. Chilling stress is particularly caused by low temperatures above the freezing point, and ice formation in plant tissues as a result of exposure to temperatures below the freezing point is known as freezing stress. In order to minimize the negative impacts of low-temperature stress on normal growth, plants undergo a plethora of physiological and biochemical adjustments (Ding et al., 2019). Studying how plants respond and adapt to low-temperature stress is of great importance not only for the fundamental understanding of environmental adaptation but also for generating stress-tolerant crop plants. The molecular regulation of plant low temperature stress has been a topic of intense research in recent years. The findings discussed in this Research Topic hold great potential for the development of resilient, cold-tolerant crop varieties that can withstand challenging environmental conditions, ultimately contributing to global food security.

In this Research Topic, Wang et al. focused on the studies of regulatory mechanisms of a basic helix-loop-helix (bHLH) transcription factor TaMYC2 in freeze tolerance in *Triticum aestivum* (wheat). *TaMYC2* was induced by both freezing temperature and jasmonates (JA). Overexpression of *TaMYC2* in *Arabidopsis* enhanced freeze tolerance. It has been established that the ICE-CBF-COR signaling pathway in plants regulates how plants acclimatize to cold stress (Wang et al., 2017). Here, the expression of ICE-CBF-COR module under the freezing temperature was enhanced in *TaMYC2* overexpression plant. In turn, the physical interaction of the key repressor of the JA signal transduction pathway TaJAZ7 with both TaMYC2 and TaICE41 was observed. These results showed that TaMYC2 regulates JA mediated freeze tolerance through TaJAZ7 in wheat and laid the foundation for improving crop tolerance to extreme temperature stress in the future. Du et al. focused on the identification of chilling related NAC transcription factor genes in *Kandelia obovata* by bioinformatic analysis. A total of 79 *KoNACs* were identified, and they were unequally distributed across all 18 chromosomes of *Kandelia obovata*. The expression levels of 19 *KoNACs* were significantly induced by cold treatment suggesting their potential role in *Kandelia obovata* chilling tolerance. These results demonstrates that KoNACs might be involved in cold stress and can be as candidate genes for the genetic engineering of *Kandelia obovata* with enhanced chilling tolerance. Genome-wide association studies (GWAS) are a powerful and ubiquitous tool for investigating multiple or complex traits related to different plant stresses (Cortes et al., 2021). Sahoo et al. identified sixteen chilling tolerance genes in Arabidopsis by GWAS. But only two of these genes, were previously identified as the cold tolerance genes. The low overlap (12.5%) between the genes identified in this GWAS with those discovered previously suggests that chilling tolerance is a complex physiological process governed by multiple genetic mechanisms. Recent technological advances from the combination of transcriptomic and metabolomic analyses have provided the possible approaches for dissecting molecular features involved in the re-adjustment of gene transcription and metabolic pathways during stress response (Jendoubi, 2021). Jiang et al. carried out a comprehensive study on the phenotype, physiology, functional substances, and gene expression of olive tree exposed to cold stress by combined transcriptomic and metabolomic analyses. The results showed that both the photosynthesis efficiency and antioxidant activity in cold-tolerant cultivar were higher than the cold-sensitive cultivar after cold treatment. The dynamic changes of gene expression and metabolites in the amino acid, glycerolipid, diterpenoid, and oleuropein metabolism pathways, as well as ubiquitination played an essential role in the cold stress responses of olive. Additionally, the building of the gene network for metabolites and ubiquitination suggested that among the various types of ubiquitination, polyubiquitination contributes largely to maintaining the physiological stability of olive under cold stress. Altogether, the results of this study shed light on the molecular mechanism underlying cold hardiness and could improve the breeding and selection of cold-resistant olive varieties in the future. Emerging evidence indicates that γ-aminobutyric acid (GABA) participates in alleviating the damage caused by low temperature stress in different plant species (Li et al., 2021). GABA can be synthesized through polyamines (PAs) pathway under stress condition. Xu et al. focused in a review paper on how GABA function and interconnect with polyamine in the regulation of low-temperature response in plants. Low temperature induces the synthesis of GABA and the exogenous application of either GABA or polyamine enhances cold tolerance in different plant species. The synthesis of GABA under low temperature is partly dependent on modifications in certain polyamines suggesting the interconnect of GABA with polyamine in cold stress. The application of GABA at low temperatures has been usually associated with the activation of the antioxidant defense system, thereby tending to indicate that GABA enhances the tolerance to low-temperature stress by modulating reactive oxygen species (ROS) content. It is necessary to study on the molecular genetic mechanisms of plant low temperature stress response, which is of great importance not only for the fundamental understanding of cold adaptation but also for generating low temperature-tolerant crop plants.

## Author contributions

BZ: Conceptualization, Supervision, Writing – original draft, Writing – review & editing. LY: Writing – review & editing. WW: Writing – review & editing. ZZ: Writing – review & editing.
